# Chromophobe Renal Cell Carcinoma with Sarcomatoid Differentiation (osteosarcomatous and chondrosarcomatous differentiation)—A Case Report and Comprehensive Review

**DOI:** 10.15586/jkcvhl.v11i3.343

**Published:** 2024-09-10

**Authors:** Meenakshi Rao, Anju G, Shiv Charan Navriya, Binit Sureka, Jeena Raju Kudunthail

**Affiliations:** 1 Department of Pathology & Lab Medicine, All India Institute of Medical Sciences, Jodhpur, Rajasthan, India;; 2 Department of Urology, All India Institute of Medical Sciences, Jodhpur, Rajasthan, India;; 3 Department of Diagnostic and Interventional Radiology, All India Institute of Medical Sciences, Jodhpur, Rajasthan, India

**Keywords:** chondrosarcomatous differentiation, chromophobe, osteosarcomatous differentiation, renal cell carcinoma

## Abstract

Chromophobe renal cell carcinomas (ChRCCs) have a good prognosis and comprise approximately 5–7% of renal cell carcinomas (RCCs). The sarcomatoid differentiation in RCC is found in around 5–10% of cases; however, in ChRCC, it is much less than in other RCCs and poorly responds to chemotherapeutic agents. A study by de Peralta-Venturina et al. found 9% sarcomatoid differentiation in chromophobe RCC. We present the case of a 58-year-old female with a left abdominal mass diagnosed as ChRCC with the existence of sarcomatous differentiation including osteosarcomatous and chondrosarcomatous, which are of adverse prognosis. Osteosarcoma-like divergent differentiation in RCC is extremely rare, with limited documented cases. It should be carefully considered in evaluating and managing renal masses due to its potential impact on clinical outcomes.

## Introduction

Chromophobe renal cell carcinomas (ChRCCs) comprise approximately 5–7% of renal cell carcinomas (RCCs). The median age of diagnosis is 59 years, from 17 to 92 years (1–4). Similar to oncocytoma, ChRCC is believed to originate from the intercalated cells within the renal cortex (2).

The majority of ChRCC tumors are sporadic. However, rare hereditary forms, such as Birt–Hogg–Dubé syndrome, are associated with mutations in FLCN, and Cowden syndrome is linked to mutations in PTEN. ChRCCs exhibit chromosomal losses affecting chromosomes 1, 2, 6, 10, 13, 17, 21, and Y and somatic mitochondrial DNA mutations (1, 2, 4, 5).

Sarcomatoid dedifferentiation can occur in any type of RCC, including clear cell carcinoma, papillary RCC, and collecting duct carcinoma, and it is confirmed when the parent tumor undergoes dedifferentiation into a higher-grade malignancy. It is characterized by its large size, necrosis, and marked cytological atypia, with highly bizarre spindle cells resembling those in sarcomas (5). However, the presence of heterologous osteosarcoma components in sarcomatoid chromophobe RCC is rare (6, 7, 8).

Before the approval of immune checkpoint inhibitors (CPIs), the primary treatment for advanced RCC involved the use of tyrosine kinase inhibitors (TKIs) targeting vascular endothelial growth factor (VEGF) or molecular inhibitors targeting the mammalian target of rapamycin (mTOR). These treatments had limited effectiveness, particularly in cases with sarcomatoid differentiation, which responded poorly to TKIs and chemotherapeutic agents (9, 10).

In this report, we present the case of a 58-year-old female with a left abdominal mass diagnosed as ChRCC, manifesting osteosarcomatous and chondrosarcomatous differentiation.

## Case Details

**Figure 1:**
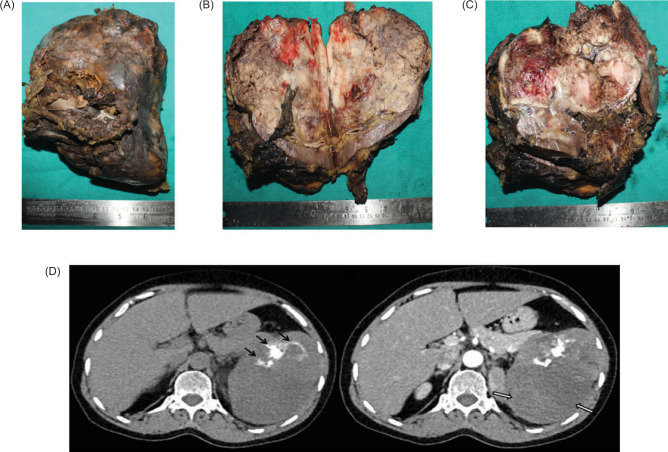
Photograph of enlarged left radial nephrectomy specimen (A); The cut surface shows a heterogeneous variegated mass in the upper and mid poles, with a grayish white to grayish brown color, firm, and large areas of necrosis and hemorrhage grossly involving the renal sinus, pelvis, and hilar area and extending till the perinephric fat (B & C); CT image reveals a large, well-defined, predominantly hypoenhancing mass lesion in the left kidney with internal coarse calcifications (D).

We present the case of a woman in her 50s who has been clinically and radiologically diagnosed with left-sided RCC. A left radical nephrectomy was performed. CT findings reveal a large, well-defined, predominantly hypoenhancing mass lesion arising from the upper and mid-pole of the left kidney with internal coarse calcifications, with differentials likely that of RCC, chromophobe, or papillary variants needing consideration (Figure 1).

On gross examination, an enlarged left radial nephrectomy specimen with a heterogeneous variegated mass was noted in the upper and mid poles, with a grayish white to grayish brown, firm cut surface, and large areas of necrosis and hemorrhage. The tumor was 14 × 13 × 9.5 cm in size, and it grossly involved the renal sinus, pelvis, and hilar area and extended to the perinephric fat (Figure 1).

**Figure 2:**
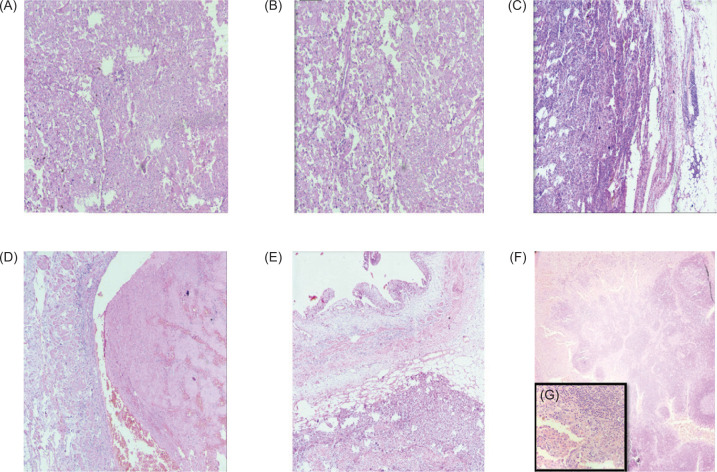
Micro pictograph shows the tumor arranged in sheets, lobules, solid nests, trabecular, alveolar, and papillaroid patterns, separated by thin, delicate fibrovascular septae (A: H&E; 100x, B: H&E; 400x ); The tumor cells infiltrate into perinephric fat (C, H&E; 200x); Microphotograph shows lymphovascular invasion (D: H&E; 400x); Microphotograph shows pelvicalyceal system invasion (E: H&E; 200x); Microphotograph shows lymph node involvement by tumor, inset high power view (F & G: H&E; 100x, 400x).

**Figure 3:**
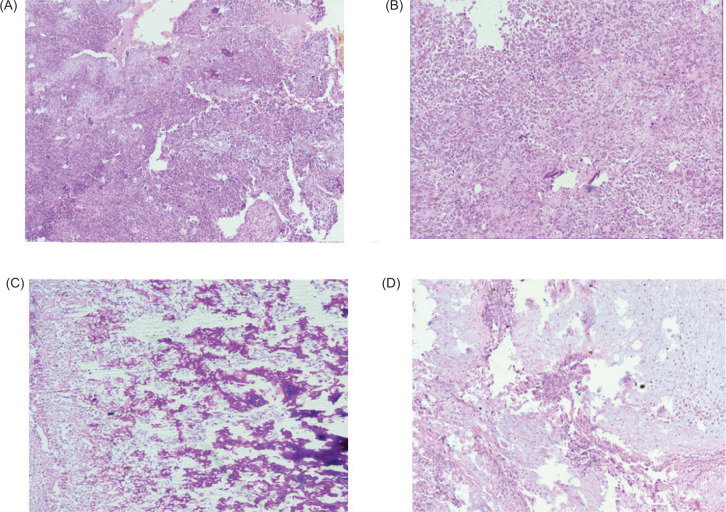
Micro pictograph shows extensive sarcomatoid differentiation (A & B: H&E; 200x, H&E 400x); Osteosarcomatous differentiation (C: H&E; 400x); Chondrosarcomatous differentiation (D: H&E; 400x).

On microscopy, the tumor was arranged in sheets, lobules, solid nests, trabecular, alveolar, and papillaroid patterns, separated by thin, delicate fibrovascular septae. The cells were large, round to polygonal, with well-defined cell membranes. They exhibited moderate nuclear pleomorphism, had abundant bright eosinophilic to focally clear to foamy cytoplasm, hyperchromatic resinoid nuclei with focal perinuclear halo, and inconspicuous to some cells showing prominent nucleoli (nucleoli conspicuous and basophilic at 400x magnification). Please refer to Figure 2. Brisk mitosis, a few large bizarre cells, and multinucleated tumor giant cells were also seen. In 70% of the viable tumors, extensive sarcomatoid differentiation is identified with osteosarcomatous and chondrosarcomatous differentiation. The cancer involves regional lymph nodes (Figures 2 and 3).

**Figure 4:**
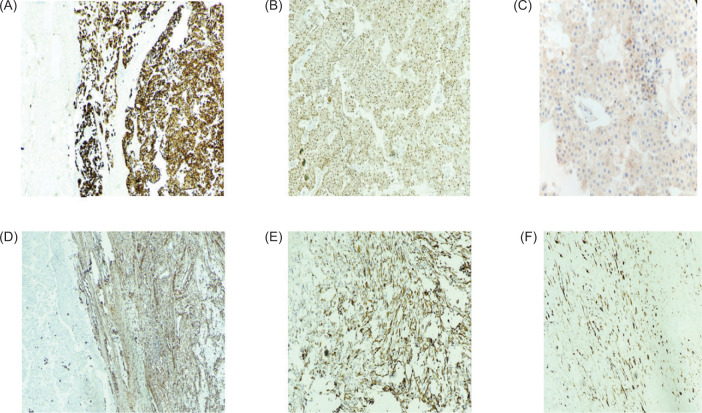
On immunohistochemistry, round to polygonal tumor cells show positivity for Cytokeratin 7 (A: 200x), PAX8 (B: 200x), and CD117 (C: 400x). The cells of sarcomatous differentiation show positive for immunohistochemical markers: vimentin (D: 200x), desmin (E), and focally positive for SMA (F; 400x).

On immunohistochemistry (IHC), the round to polygonal tumor cells were positive for PAX8, CD117, and CK7, showed retained INI-1 expression, and were negative for vimentin and AMACR. Morphological with IHC features confirm a diagnosis of ChRCC Grade 4, with 70% sarcomatoid, osteosarcomatous, and chondrosarcomatous differentiation. The cells with sarcomatous differentiation showed immunoreactivity for immunohistochemical markers, vimentin, CD117, desmin, and focally for smooth muscle actin (SMA). Please refer to Figure 4.

## Discussion

Chromophobe renal cell carcinoma generally has a better prognosis compared to clear cell RCC (ccRCC) or papillary RCC, with a 5-year survival rate ranging from 78 to 100% (2).

Sarcomatoid differentiation is present in ChRCC, constituting 9% of all RCC subtypes. In a study done by de Peralta-Venturina et al., analysis of 100 kidney tumors revealed an 8% rate of sarcomatoid differentiation in conventional RCC, 9% in ChRCC, 3% in papillary RCC, 29% in collecting duct carcinoma, and 11% in unclassified RCC (6, 11, 8).

Chromophobe RCC is characterized by chromosomal losses (6, 12, 3, 8, 13), leading to hypodiploid cells. These hypodiploid cells can potentially undergo polyploidization by doubling their chromosome count, initiating genetic alterations responsible for sarcomatous transformation (7, 14). In literature, Tun et al. reported that RCC cells exhibit stem cell-like properties via epithelial–mesenchymal transition (EMT). Additionally, they demonstrated in vitro osteogenic and adipogenic differentiation capabilities in RCC cells by EMT (15).

Jones et al. documented monoclonal cell proliferation in epithelial and sarcomatoid elements (16). Bi et al. reported that 43% of somatic mutations are shared between carcinomatous and sarcomatous elements, suggesting a common clonal precursor (13). Wang et al. proposed that sarcomatoid RCC is a heterogeneous group that differentiates based on the underlying parent subtype rather than being a singular entity. Specifically, sarcomatoid ccRCC, clinically aggressive, appears to have distinct tumorigenesis (17).

The literature review describes only a limited number of documented ChRCC cases with sarcomatoid differentiation. However, cases with chondrosarcomatous and osteosarcomatous differentiation are sparse. Hyodo et al. reported a case of ChRCC with sarcomatoid differentiation, including mainly chondrosarcoma, some osteosarcoma, and a lipomatous area (18). Both Quiroga-Garza et al. (19) and Hes et al. (20) reported sarcomatoid ChRCC cases featuring heterologous sarcomatoid elements

In recent years, the advent of immune checkpoint inhibitors (CPIs) has led to improved outcomes for patients with metastatic RCC. Nivolumab, a fully human monoclonal IgG4 antibody targeting programed cell death-1 (PD-1), and ipilimumab, a recombinant human IgG1 antibody that binds to cytotoxic T-lymphocyte-associated antigen 4 (CTLA-4), have both demonstrated therapeutic efficacy in the treatment of RCC (9, 10).

A recent study showed that RCC with sarcomatoid differentiation expresses PD-L1 at higher levels than RCC without sarcomatoid differentiation. This finding suggests that patients with sarcomatoid RCC may be good candidates for treatment with PD-1/PD-L1 blockers (9, 21).

Although the current treatment guidelines from the National Comprehensive Cancer Network (NCCN) do not differentiate RCC based on the presence of a sarcomatoid component, immune CPIs should be considered early in the treatment (22).

The patient was under follow-up for 5 months and received two cycles of chemotherapy (sunitinib) following a nephrectomy. During treatment, she had severe pain due to multiple metastases. Following that, her condition deteriorated, and she unfortunately passed away. Due to economic constraints, immune CPIs could not be initiated in this case.

The occurrence of osteosarcoma-like divergent differentiation in RCC is exceedingly rare, with only a few reported cases known to date (7, 1,8–20, 23).

## Conclusion

In summary, RCC with sarcomatoid transformation leads to a poorer prognosis. Osteosarcoma-like differentiation within RCC is rare, documented only in a few cases. Recognition is vital for accurately diagnosing and managing renal masses despite their rarity. Early identification enables treatment and optimizes patient outcomes. A multidisciplinary approach involving pathologists, radiologists, and clinicians is crucial. Cases with sarcomatoid osteoid and chondroid differentiation underline the necessity for histological assessment in guiding optimal patient management. Upcoming CPIs such as PD-L1 blockers can change the prognosis, as sarcomatous differentiation had a poorer outcome than RCC without sarcomatous differentiation.
